# Role of inflammation, immunity, and oxidative stress in hypertension: New insights and potential therapeutic targets

**DOI:** 10.3389/fimmu.2022.1098725

**Published:** 2023-01-10

**Authors:** Zenglei Zhang, Lin Zhao, Xingyu Zhou, Xu Meng, Xianliang Zhou

**Affiliations:** Department of Cardiology, Fuwai Hospital, National Center for Cardiovascular Diseases, Chinese Academy of Medical Sciences and Peking Union Medical College, Beijing, China

**Keywords:** hypertension, inflammation, immunity, oxidative stress, cytokines, cardiovascular diseases

## Abstract

Hypertension is regarded as the most prominent risk factor for cardiovascular diseases, which have become a primary cause of death, and recent research has demonstrated that chronic inflammation is involved in the pathogenesis of hypertension. Both innate and adaptive immunity are now known to promote the elevation of blood pressure by triggering vascular inflammation and microvascular remodeling. For example, as an important part of innate immune system, classically activated macrophages (M1), neutrophils, and dendritic cells contribute to hypertension by secreting inflammatory cy3tokines. In particular, interferon-gamma (IFN-γ) and interleukin-17 (IL-17) produced by activated T lymphocytes contribute to hypertension by inducing oxidative stress injury and endothelial dysfunction. However, the regulatory T cells and alternatively activated macrophages (M2) may have a protective role in hypertension. Although inflammation is related to hypertension, the exact mechanisms are complex and unclear. The present review aims to reveal the roles of inflammation, immunity, and oxidative stress in the initiation and evolution of hypertension. We envisage that the review will strengthen public understanding of the pathophysiological mechanisms of hypertension and may provide new insights and potential therapeutic strategies for hypertension.

## 1 Introduction

Hypertension has become the leading global risk factor for cardiovascular diseases (CVDs) (1), and 1.15 billion adult individuals have been confirmed with hypertension according to related data (2). In addition, the American Heart Association and the American College of Cardiology formulated strict new diagnostic criteria that are expected to extend the number of patients with hypertension by two–threefold (3, 4). Many common and effective drugs, including angiotensin-converting enzyme inhibitors (ACEIs)/angiotensin II (Ang II) type 1 receptor blockers (ARBs), beta-blockers, calcium channel blockers (CCBs), and diuretics, have been widely confirmed to control raised blood pressure (5), causing a significant reduction of mortality associated with hypertension; however, hypertension still remains a major public health problem and health care burden in both developed and developing countries. Blood pressure is still poorly controlled in approximately half of patients who are receiving treatment (6), so potential therapeutic targets are urgently needed.

The pathogenesis of hypertension is complex and there are many risk factors. Accumulating evidence confirms that an activated inflammatory response and immune system are an indispensable part in the genesis and evolution of hypertension and are associated with hypertensive complications, such as myocardial infraction, hemorrhagic stroke, and renal injury (7). The elevated level of inflammatory biomarkers, including C-reactive protein (CRP) and cytokines, have been detected in patients with hypertension, revealing that the immune system is involved in hypertension as a low-grade inflammatory condition which is a chronic and continuous process and is more characterized by the increase of inflammatory cells and inflammatory mediators compared with infectious diseases, and usually does not show significant symptoms (5, 8, 9). Elevated BP by many risk factors including genetic susceptibility and several environmental factors can induce organ injury promoting the formation of damage-associated molecular patterns (DAMPs) and new antigens which are regarded as the main impetus for the low-grade inflammation (10). Inappropriate immune activation may act in the kidney, microvascular, nervous system, and even gut microbiome to promote the elevation of blood pressure (11–17), especially salt-sensitive hypertension (18, 19). Inflammation is generally considered an immune response and thus, in the early stage, mainly engages the innate immune system (IIS) and is subsequently followed by the adaptive immune response. Although interactions occur between these responses, they have different effects on disease progression and clinical significance; therefore, we will discuss these separately in this review.

In 1967, Okuda and Grollman first reported there was a relationship between the immune and vascular systems based on the induction of artery hypertension in rats after they had received lymphocytes derived from rats with hypertension (20). According to the surface markers and functions, macrophages are classified into proinflammatory M1 macrophages and anti-inflammatory M2 macrophages. The recent study has confirmed the damage effect of M1 macrophages and the inconsiderably protective role of M2 macrophages in hypertension (10). Moreover, increasing evidence showed that hypertension has a chronic inflammatory status characterized as the transmigration, accumulation, and activation of inflammatory cells, and the proinflammatory cytokines and free radicals produced by activated innate immune cells and endothelial cells (21). In the present review, we will discuss how the immune system and associated inflammation and oxidative stress affect the procession of hypertension and will summarize the potential therapeutic targets in hypertension.

## 2 The relationship of inflammation, immunity, and oxidative stress

Although numerous risk factors promote the genesis and progress of hypertension, the role of inflammation, immunity, and oxidative stress have been overwhelming confirmed by evidence from many laboratories worldwide (22–26). The causal state of circulating immune cells, such as monocytes, neutrophils, and lymphocytes, has been demonstrated (27), and the imbalanced activated immune system is known to produce an inflammatory condition with an increasing amount of proinflammatory cytokines. First, leukocytes gather *via* cytokine and chemokine signaling and roll on the vascular endothelium regulated by E and P selectin in the early phase of vascular inflammation. The interaction of leucocyte integrins and the intracellular adhesion molecules (ICAMs) 1–5 and vascular cell adhesion molecule 1 (VCAM-1) then play a crucial part in the subsequent processes (28). Cytokines and chemokines promote oxidative stress, a typical characteristic of essential hypertension. Reactive oxygen species (ROS) are the major effector of the oxidative stress injury and consequently may have a crucial role in connecting inflammation, immune system, and hypertension (10). A low inflammatory condition, resulting from massive production of the superoxide anion (·O_2_) and hydrogen peroxide (H_2_O_2_) by endothelial cells, monocytes, and macrophages, promotes oxidative stress and causes vascular dysfunction and target-organ damage in the process of hypertension (29). The activated renin-angiotensin-aldosterone system (RAAS) is a crucial regulator of oxidative stress, and results in the inflammatory damage of vessels (30). As the major component of the RAAS, Ang II promotes vascular inflammation by activating nicotinamide adenine dinucleotide phosphate oxidase oxidases (NOXs) and increasing the expression of endothelin-1 (ET-1), causing the production of a large number of proinflammatory mediators that contribute to the endothelial dysfunction. The main function of NOXs are producing ROS. For example, the low level of ROS produced by NOX2 in physiological state is closely related to the process of cell proliferation and differentiation. However, excessive ROS by activated NOXs is responsible for the CVDs, especially hypertension. Although 7 isoforms of NOXs have been reported, NOX2 and 4 are considered as having tight association with CVDs. NOX 2 and 4 are mainly expressed in endothelial cells and cardiomyocytes (31). NOX2 causes vascular oxidative stress *via* producing superoxide directly, while NOX4 mainly depend on the production of H_2_O_2_ through the rapid dismutation of superoxide into H_2_O_2_ (32). The role that activated NOX2 contributes to hypertension *via* mediating oxidative stress injury and further promoting endothelial dysfunction has been observed (33, 34). According to the results from Toral M et al., the NOX2 inhibitor decreased vascular ROS production and restored endothelial dysfunction in the hypertensive mice (34). NOX4 facilitates vascular hyperproliferation and microvascular remodeling through the induced hyperoxidation and ER stress (35). Furthermore, Ang II, ET-1, and aldosterone may be responsible for impaired remodeling of vessels in a large extent *via* upregulating expression of chemokines in vascular smooth muscle cells and endothelial cells (36, 37). In addition, activated neutrophils can release ROS as well as proinflammatory cytokines, causing significant oxidative stress. The positive association between the concentration of CRP and the level of oxidative stress has been confirmed (38), and Savoia et al. reported that CRP may upregulate expression of angiotensin type 1 receptors, which could then modulate the formation of ROS (39).

The kidneys have an indispensable role in modulating blood pressure, and renal hypertension resulting from chronic kidney injury and renal artery stenosis is one of most common secondary hypertension diseases globally (40). ROS has been detected in the early stage of chronic kidney disease and exasperates renal function contributing to oxidative stress and chronic inflammation (41, 42), which further accelerates the process of hypertension.

In summary, oxidative stress may be a trigger as well as the result of both inflammation and imbalance in the immune system, contributing to the vascular injury and remodeling, and results in the progression of hypertension.

## 3 Pathophysiological mechanisms of essential hypertension

Essential hypertension is a disease of unknown etiology that includes the complex interaction of many factors, especially those of genetic and environmental origin. However, it is unclear how these factors increase blood pressure. Increasing evidence indicates that essential hypertension is not a homogeneous disease and that the etiology and pathogenesis vary among individuals. Furthermore, hypertension has a long course and generally slow progression. The mechanisms of initiation, maintenance, and acceleration are different in different stages, and there are interactions among various pathogenic mechanisms (e.g., oxidative stress and inflammation and the immune system) (43). Therefore, hypertension is a multi-factor, -link, and -stage disease that is also affected by differences between individuals. In this section, we intend to briefly summarize the potential pathophysiological process of essential hypertension.

The main mechanisms of hypertension include microvascular remodeling, imbalance of the autonomic nervous system (ANS), and activation of the RAAS (43). The increase of peripheral resistance in small arteries, ranging from 100 to 300 µm in diameter, is the central mechanism and the most significant characteristic in essential hypertension (44). As mentioned above, ROS, activated immune cells, and inflammation, can stimulate endothelial cells to produce and release large amounts of vasoactive substances, such as nitric oxide (NO), prostaglandin-I-2 (PGI2), ET-1, and endothelium-dependent vasoconstrictor factor (EDGF) (44). Age and various cardiovascular risk factors, including dyslipidemia, elevated blood sugar, and smoking, lead to abnormal blood endothelial cell function, increase the production of oxygen free radicals, enhance NO inactivation, vascular inflammation, and oxidative stress response, and influence the elastic function and structure of arteries. With the decreased elasticity of the aorta, the pulse wave conduction velocity increases, and the phase of the reflected wave arriving at the central aorta advances from diastole to systole. The occurrence of a delayed systolic pressure peak can elevate systolic blood pressure and reduce diastolic blood pressure. Changes in the structure (sparse number of tubes or increased wall/lumen ratio) and function (decreased elasticity and increased resistance) and function of resistance arterioles affect peripheral pressure responses.

The position of the origin or the intensity of the reflected wave also has an important part in increasing the pulse pressure. In the nervous system, various reasons can cause changes in the function of the cerebral subcortical nerve center leading to abnormal concentrations and activities of various neuropeptides and neurotransmitters, including norepinephrine, epinephrine, dopamine, serotonin, vasopressin, enkephalin, and brain natriuretic peptide as well as changes in the central renin-angiotensin system. These changes eventually hyperactivate the sympathetic nerve system, increasing the concentrations of plasma catecholamines and constriction of arteries and causing resistance and increased blood pressure (45, 46).

AT-II is the main effector of the RAAS and is involved in a range of processes that can increase blood pressure. These include acting on angiotensin receptor 1, making arteriole smooth muscle contract, stimulating the glomerular zona of adrenal cortex to secrete aldosterone, and promoting the release of norepinephrine through the positive feedback in sympathetic nerve terminal presynaptic membrane. Recently, many tissues, including the blood vessel wall, heart, central nervous system, kidney, and adrenal gland, have been found to contain various components of the RAAS (47). The role of tissue RAAS in the function and structure of the heart and blood vessels may have a greater impact on the occurrence and maintenance of hypertension (44). In addition, water and sodium retention and insulin resistance can contribute to elevating blood pressure.

## 4 Inflammation in hypertension

Inflammation is a rapid, nonspecific defense response of the body that acts to maintain hemostasis *via* monitoring and clearing of foreign bodies. This involves coordinating vascular endothelial cells and circulating inflammatory cells and cytokines (48). Although inflammation has beneficial effects, including eradicating pathogens and protecting organs from damage, imbalanced regulation may cause serious and sustained inflammatory response that can in turn cause progressive tissue injury, organ dysfunction, and fibrosis, and even systemic inflammatory response syndrome. Overwhelming evidence shows that low-grade chronic inflammation contributes to the initiation and maintenance of essential hypertension (49, 50). A recent study reported that the chronic inflammatory condition induced by excessive and prolonged stimulation of IIS could lead to vascular endothelial cell damage (51). In this section, we mainly discuss the role of inflammasomes and inflammatory cytokines and of neuroinflammation in the progress of essential hypertension.

### 4.1 Inflammasomes

**Figure 1:**
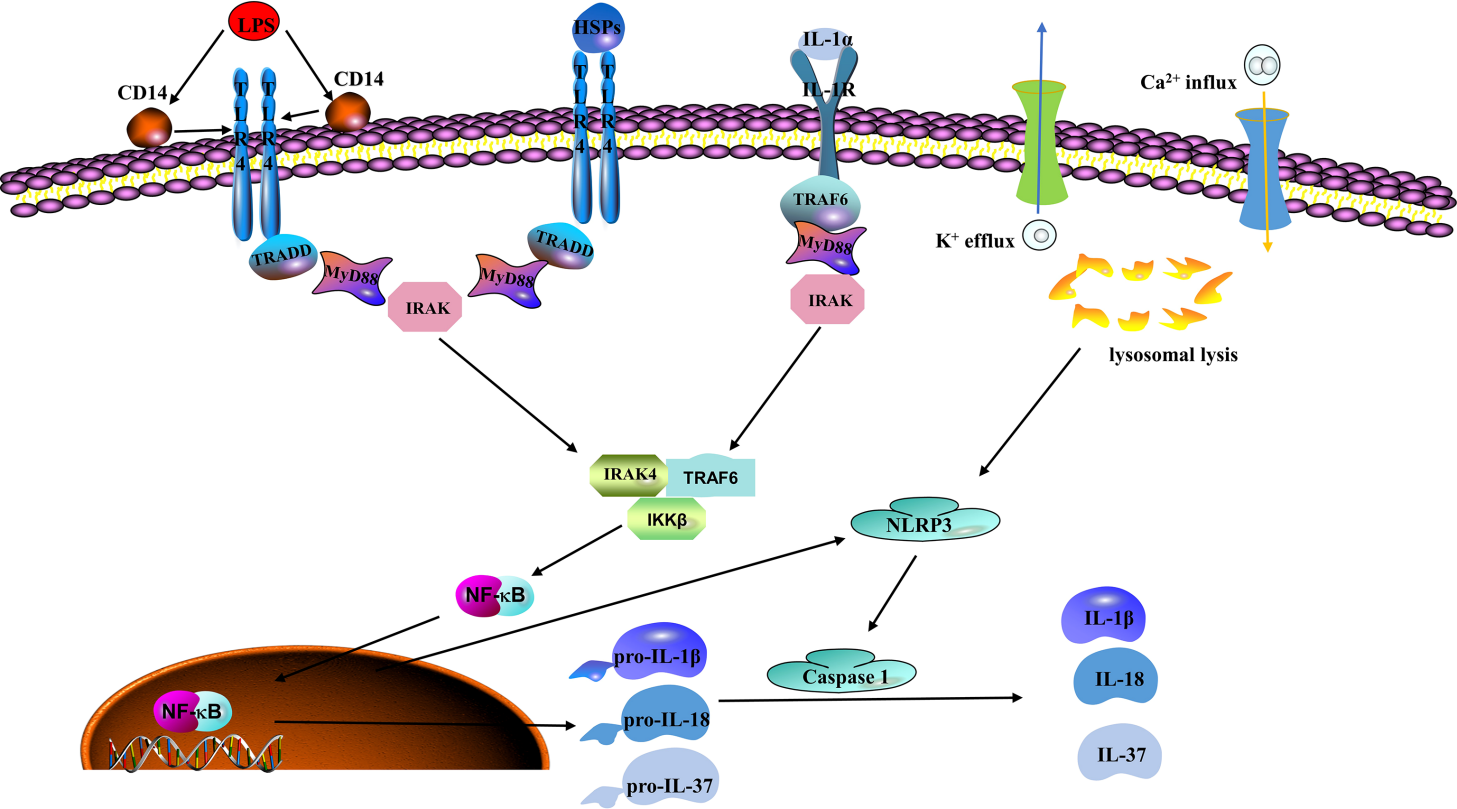
Classic mechanisms of inflammasome activation. Lipopolysaccharide (LPS), which is regarded as the prototype of pathogen associated molecular patterns (PAMPs) and heat shock proteins (HSPs) as a typical representative of damage associated molecular patterns (DAMPs), can be recognized by toll-like receptor 4 (TLR4). Nuclear factor kappa B (NFκB) mediates these signals to produce NOD-like receptor family pyrin domain containing 3 (NLRP3), pro-IL-1β, pro-IL-18, and pro-IL-37. Next, NLRP3 is activated by potassium (K+) efflux, calcium (Ca2+) influx, and lysosomal leakage to recruit cysteine-requiring aspartate protease-1 (caspase-1). Caspase-1 then induces the maturation and release of pro-IL-1β, pro-IL-18, and pro-IL-37 and other related inflammatory agents that cause the inflammatory reaction and participate in the occurrence and development of the disease.

The inflammasomes are cytosolic protein complexes, such as NLR-family pyrin domain-containing protein (NLRP)1 and NLRP3 mainly expressed in innate immune cells (like monocytes and macrophages) and endothelial cells, that identify pathogens and activate inflammatory responses, including the intracellular IIS receptors (52, 53). As inflammasomes are major components of the IIS, they mediate important inflammatory responses and pyroptosis, which are tightly associated with endothelial dysfunction. Improperly activated inflammasomes are involved in the potential pathogenesis of many inflammations related diseases (54). NLRP3 is the most characteristic inflammasome among the pattern recognition receptors (PRRs) that recognize damage-associated molecular patterns (DAMPs) and pathogen-associated molecular patterns (PAMPs) to initiate and promote an inflammation response (55). Briefly, a wide range of DAMPs or PAMPs stimulate NLRP3 activation by inducing ion transporting (e.g., K^+^ efflux and Ca^2+^ influx) and lysosomal leakage. The activated NLRP3 inflammasome then triggers caspases to induce cleavage of inactive proinflammatory cytokine precursors, such as pro-IL-1β, pro-IL-18, and pro-IL-37 (**Figure 1**) (48). Numerous studies show that activated NLRP3 inflammasomes are tightly associated with many chronic inflammatory and metabolic diseases, such as ischemic heart diseases and diabetes mellitus, stroke, atherosclerosis, and hypertension (56–59). Studies have suggested that inflammasomes and their related cytokines are tightly associated with raised blood pressure. According to Dalekos et al., an elevated serum concentration of IL-1β was detected in patients with hypertension (60). Endothelial cells are not only the action target of IL-1β but also produce IL-1β (61). *In vitro* experiments show that monocytes derived from hypertensive patients can release a high level of IL-1β in response to the stimulation of Ang II or lipopolysaccharide (LPS) (62). In addition, the production of mitochondrial ROS is a vital component of cellular oxidative stress, and NLRP3 activation is indispensable in this process. More and more agonists have been reported to produce vascular inflammation and injury by triggering NLRP3 inflammasome activation. According to Xie et al., the NLRP3 inflammasome is involved in visfatin-mediated vascular injury, which may cause atherosclerosis (63). IL-1β and IL-18 would then be released into the blood flow to activate more inflammatory cells and thereby expand the inflammatory response when inflammasomes are activated.

Recent research suggests that endothelial dysfunction is associated with activated NLRP3 inflammasomes through the release of HMGB1. Activated NLRP3 inflammasome and subsequent release of HMGB1 have been considered as the underlying cause of cell-to-cell junction breaks in mouse vascular endothelial cells treated with high-glucose levels (64). Interestingly, NLRP3 deficiency has a significantly protective role *via* preventing tight junction disruption in diabetic mice and ameliorating endothelial permeability in endothelial cells *in vitro* (64), and inhibiting the NLRP3 inflammasome by pharmacological inhibition also produced potent therapeutic effects (65). More recently, knockdown of NLRP3 also exhibited remarkable effect on lowering blood pressure, improving vascular remodeling and insulin resistance, and ameliorating or delaying the atherosclerosis *via* regulating metabolism, relieving oxidative stress and reducing release of inflammatory cytokines (58, 66, 67). WEHD, a caspase-1 inhibitor, could significantly reduce inflammasome activity and prohibited ATP-induced hypertension in mice (68).

### 4.2 Role of cytokines in raising blood pressure

Traditionally, essential hypertension was thought to be caused by hemodynamic changes, but in recent years, numerous studies have revealed that inflammatory cytokines play a significant part in promoting the progression of hypertension *via* affecting vascular and renal function. Cytokines are a major component of the immune system and the main performer of inflammatory response, which connects them with vascular remodeling and hypertension. Until recently, the connection between how inflammation and immunity affect blood pressure levels was not well understood. According to previous observations, numerous T cells and monocytes/macrophages accumulated in vessels and kidneys in the mice model with renal ischemia-reperfusion injury (69). Based on other’s and our own research, these cells have been shown to secret potent cytokines that impair organs, especially the heart and kidneys. Modern experimental technologies have demonstrated that a variety of cytokines are involved in the initiation and progression of hypertension (**Table 1**).

**Table 1:** Examples of inflammatory cytokines involved in hypertension.

Cytokines	Cell sources	Disease models	Mechanisms	Biological effects	Reference
IL-17	CD4^+^ T cells and some CD8^+^ cells	IL-17A^-/-^ mice	Enhance CCL-2 in an NADPH oxidase-dependent manner, and altering gene expression in vascular smooth muscle cells	Promotes oxidative stress and increases the number of infiltrated T cells	(70–72)
IL-6	Monocytes, macrophages, dendritic cells, etc.	IL-6^-/-^ mice	polarizes the CD4^+^ cells and facilitates water and sodium retention	promotes the production of IL-17 and upregulates the expression and increase the activity of the epithelial sodium channel in duct cells	(73–75)
TNF-α	Monocytes and macrophages	rheumatoid arthritis patients	NF-κB and NADPH oxidase activation	upregulates the expressions of chemokine and adhesion molecule in vessels, facilitates microvascular remodeling and sodium retention, and reduce NO production	(76–81)
IFN-γ	Th1 cells	Ang II-treated IFN-γR knockout mice	Increases the angiotensinogen expression	Promotes renal fibrosis and decreases glomerular filtration rate	(82)
IL-1β	Monocytes, T cells and neutrophils	Diabetic db/db mice	Polarize the naïve macrophages into M1 subtype	Releases a large amount of IL-6	(83)

CCL-2, C-C motif chemokine ligand 2; IL, interleukin; IFN-γ, interferon gamma; NADPH, nicotinamide adenine dinucleotide phosphate; NF-κB, nuclear factor kappa-B; NO, nitric oxide; Th, T helper; TNF-α, tumor necrosis factor α.

#### 4.2.1 Interleukin 17

IL-17 is derived from TH17 cells, an important subset of CD4^+^ T cells, and includes several isoforms, including IL-17A and IL-17F; several B cells and CD8^+^ cells also produce IL-17 (84). IL-17A is generally considered to act synergistically with other cytokines (e.g., TNF) during inflammatory response. IL-17A and TNF-α have been demonstrated to synergistically enhance lung inflammation and expression of C-C motif chemokine ligand 2 (CCL2) in an NADPH oxidase-dependent manner (70), which affects vascular functions by influencing gene expression (71). Moreover, IL-17A is reported to be involved in multiple inflammatory diseases, such as asthma, psoriasis, and systemic lupus erythematosus (84). Considering the important position of IL-17 in inflammation-related diseases, research on anti-IL-17 monoclonal antibodies (Ixekizumab and Secukinumab) and anti-IL-17 receptor antibodies (Brodalumab) has become a scientific hotspot in recent years, and these antibodies have shown satisfactory treatment effects in clinical trials (85, 86).

Growing evidence has linked IL-17A to increased blood pressure, although the exact mechanisms are unclear. Plasma IL-17A concentrations were significantly increased in Ang II-induced animal models with hypertension. In contrast, Ang II infusion could not impair endothelial function or result in an elevation of blood pressure in IL-17A^-/-^ mice. A similar result was observed when the level of oxidative stress was lowered and infiltration of T cells decreased in IL-17A^-/-^ mice compared with that in the control group. Treatment with anti-IL-17 antibody was found to significantly lower blood pressure and reduce collagen deposition in mouse heart and kidneys. Consistent with these findings, when co-cultured human vascular smooth muscle cells (VSMCs) with TNF-α *in vitro*, IL-17A facilitate the expression of various cytokines and chemokines, such as CCL7 and 8, CXCL2 and CSF3, which could recruit more inflammatory cells. Subsequently, another exact role of IL-17A in hypertension has been demonstrated by Nguyen et al. that IL-17A could reduce the production of NO by phosphorylating threonine 495 of endothelial nitric oxide synthase (eNOS) in porcine aortic endothelial cells (87). And the study also showed intravenous administration of IN-17A could elevate the blood pressure modestly in the normal mice. Taking together, inhibition of IL-23/IL-17 axis has been regarded as promising therapeutic target in the future precise therapy of hypertension (72).

#### 4.2.2 Interleukin 6

IL-6 is a 21 kDa glycoprotein secreted by monocytes, macrophages, and dendritic cells, which can activate related genes and upregulate expression of receptors involved in cell proliferation, differentiation, and apoptosis. Once the receptor recognizes the IL-6 signals, the related cellular events, such as activation of Janus kinases and Ras-mediated signal pathways, are initiated immediately (88). IL-6 is responsible for regulating the expression of the acute-phase plasma proteins in liver cells. In addition, IL-6 could facilitate the production of IL-17 *via* polarizing CD4^+^ T cells. Mounting evidence indicates that IL-6 has a crucial role in aspects of the chronic inflammatory response, such as hypertension, rheumatoid arthritis, and ischemic heart disease. Blocking IL-6-related signaling pathways by using the human monoclonal antibody Tocilizumab has shown considerable clinically therapeutic effect on rheumatoid and juvenile arthritis, and related clinical trials are being performed in other inflammatory diseases (89). Therefore, IL-6 may well have a potential therapeutic role in CVD treatment, especially with hypertension.

Accumulating evidence strongly indicates that IL-6 signaling pathways are a vital link in Ang II-induced hypertensive animal models, as the level of IL-6 had a positive relationship with blood pressure and was significantly lower when the Ang-II receptor was blocked (73). *In vivo* animal studies show that injection of Ang II can increase plasma IL-6 concentration and blood pressure, whereas the blood pressure-raising effect of Ang-II was clearly weakened in IL-6^-/-^ mice (74, 75), and was also blocked by spironolactone, suggesting the activation of mineralocorticoid receptors. In addition, evidence from *in vitro* experiments indicated that IL-6 promotes the expression and activity of sodium channels in mouse cortical collecting duct cells (90), implicating that IL-6 may have the ability to facilitate water and sodium retention *in vivo*. Thus, these research suggested that IL-6 may a potential and promising therapeutic targets in lowering blood pressure leading to the inhibition of the activity of mineralocorticoid receptors and sodium channels in duct cells.

#### 4.2.3 Tumor necrosis factor-α

TNF-α is a well-established inflammatory cytokine in the acute phase response, whose expression has been shown to increase in human and rodent hypertension studies (91). The role of TNF-α signaling in modulating many secondary inflammatory processes, such as cytokine secretion, cell differentiation, and apoptosis, is complex and cell-type and dose-dependent (92). Clinical trial data show that elevated plasma IL-6 concentrations are tightly associated with the severity of refractory hypertension and the six-year risk of death (93). In contrast, TNF-α inhibitors could lower the level of blood pressure in patients and animal models (76, 77). Although a variety of cells can secrete TNF-α, which can activate other immune cells through TNF-α receptors (TNFR1 and TNFR2), it is mainly secreted by monocytes and macrophages. The activation of TNF-α receptors is responsible for cell apoptosis, NADPH oxidase activation, and nuclear factor kappa B (NFκB) activation (78). An accumulating body of studies suggest that NFκB and NADPH oxidase activation promote hypertension by upregulating the expression of chemokines and adhesion molecules in blood vessels, facilitating microvascular remodeling and sodium retention (79). Superoxide production from NADPH oxidase could react with endothelial NO to form the strong oxidant peroxynitrite. This can severely affect vasodilation and cause a significant elevation of blood pressure because of the decrease in NO levels. Moreover, TNF-α can affect the promoter of eNOS and help destabilize the eNOS mRNA structure, which eventually leads to the massive degradation of eNOS and reduction of NO synthesis (80, 81). In kidneys, TNF-α can inhibit eNOS activity in the renal medulla in a Rho-kinase dependent fashion, thereby reducing production of NO which inhibits sodium reabsorption at several sites along the renal tubule (94, 95), and can result in renal injury directly which shift the pressure natriuresis curve to promote the elevation of blood pressure. Thus, TNF-α plays an important role in elevating blood pressure. Unfortunately, TNF-α inhibitors are currently mainly used to treat autoimmune diseases, and there is no evidence that they can be used to lower blood pressure and improve cardiovascular prognosis, but that is worthy to explore and evaluate in the future.

#### 4.2.4 Interferon gamma

IFN-γ is the only known member of the type II family of interferons and is mainly produced by T helper (Th) 1 cells. IFN-γ is not only an important part of adaptive immune responses but also indispensable in protecting hosts from infection. William et al. reported that IFN-γ-producing CD4^+^ and CD8^+^ T cells are consistently increased in hypertensive mice (21), but, according to the results from Ishimitsu et al., subcutaneous injections of IFN-γ could lower blood pressure and alleviate proteinuria and glomerular injury in Dahl salt sensitive rats (96). However, the effect on lowering blood pressure did not observe in the mice with essential hypertension (96). The study neglected the role of endogenous IFN-γ in hypertension, which might be responsible for the conflicting results. The previous study suggested that IFN-γR deficiency can significantly attenuate ventricular hypertrophy and ventricular electrical remodeling (82). Although high doses of angiotensin were injected, blood pressure levels did not increase significantly, but the degree of renal fibrosis was reduced while the glomerular filtration rate was maintained in mice lacking the IFN-γ receptor 1 compared with that in wild-type mice (82). One of the most important mechanisms whereby IFN-γ triggers hypertension is that IFN-γ can increase the expression of angiotensinogen of rat renal proximal tubule cells in a JAK2/STAT3-dependent manner (97), which consequently increases blood volume. Therefore, it is reasonable to infer that interferon-secreting T cells may establish a link with RAAS by regulating the production of angiotensinogen, resulting in an increase in blood pressure.

#### 4.2.5 Interleukin 1 beta

IL-1β is known as an important component of the IL-1 family of interleukins and mainly derived from monocytes, T cells and neutrophils. A growing amount of evidence has confirmed the association between IL-1β and hypertension (98). Specifically, it has been demonstrated that IL-1β could upregulate many proinflammatory genes, including IL-6, IL-17 and IFN-γ, resulting in further tissue injury and inflammation related events, like hypertension and myocardial infarction (99–101). High levels of IL-1β have been detected in the serum of patients with essential hypertension in recent studies (102), indicating the role of IL-1β in elevating blood pressure. Considering the tight association with hypertension and the essential role in inflammation, more and more studies on how the IL-1β involves in pathophysiological mechanisms of hypertension have been performed and explored. According to a previous study, IL-1β not only triggers the inflammatory response directly but also mediates the phenotype and functions of VSMCs and eventually leads to vascular remodeling on inflammatory-dependent or independent mechanisms (103). A recent study which aimed to evaluate the effect of an IL-1R1 receptor Inhibitor (Anakinra) on lowering the blood pressure in patients with obesity suggested Anakinra could significantly lower the systolic blood pressure and peripheral vascular resistance (104), which supported that the IL-1β could affect the progression of hypertension. Another study suggests that IL-1β derived from renal tubular epithelial cells of diabetic db/db mice could polarize the naïve macrophages into M1 subtype which releases a large amount of IL-6 resulting in salt-sensitive hypertension, but could be blunted when inhibited the synthesis of IL-1β or knocked out the IL-1 R1 in immune cells (83). Thus collectively, IL-1β plays a crucial role in the progression of essential hypertension and may be a novel promising therapeutic targets of hypertension.

### 4.3 Gut microbiota and neuroinflammation in hypertension

Recently, we have gained greater understanding of the role of gut microbiota and neuroinflammation in the pathogenesis of hypertension. Interaction between the gut microbiota and epithelial cells of the gut-brain axis is involved in regulating ANS activity to control blood pressure. The endocrine pathway of hypertension mainly involves the activation of the hypothalamus-pituitary-adrenal axis. The complex interactions of immune-regulatory organs such as bone marrow, gut, and spleen, which can enhance vascular tone and contraction, can contribute to increases in peripheral resistance and blood pressure.

In addition, increased neural activity may result in neuroinflammatory events, activating microglia, producing more proinflammatory molecules, and an inflammatory environment in autonomic brain regions. Disorder of the gut-brain axis, including gut microbiota dysbiosis, gut epithelial injury, and deranged brain input, is responsible for hypertension through inflammatory mediators, metabolites, circulating bacteria, and altered afferent information, causing neuroinflammation and disorder of the ANS. This in turn can negatively impact gut functions and associated microflora to create a vicious spiral. In this section, we address the role of an impaired gut-brain axis in the pathophysiology of hypertension.

The ANS can regulate the arterial blood pressure by controlling the vasomotor activity of sympathetic and parasympathetic nerves. Numerous inflammatory cytokines, including IL-1β (105), TNF-α, IL-6 (88), and IFN-γ (91), were detected in animal brains in hypertension models, and these cytokines have been demonstrated to raise blood pressure by increasing sympathetic nerve activity. Furthermore, the impaired blood-brain barrier is another important cause of neuroinflammation. For example, increasing evidence suggests that Ang-II as well as gut microbes and their metabolites affect the blood-brain barrier permeability, which allows more cytokines to enter the brain. Once the blood-brain barrier is disturbed, circulating inflammatory factors and toxins can enter autonomic brain regions, interfering with normal neural activity and causing hypertension (106). Microglial cells are the important part of the IIS in the brain, and responsible for homeostasis of the central nervous system *via* clearing senescent and apoptotic cells (107). Inhibition of microglia activation helps to decrease hypertension and alleviate sympathetic activation and peripheral inflammation (108). Interestingly, specific deletion of microglia could significantly relieve the Ang II-induced elevation of blood pressure and inflammation (109).

In recent years, the gut microbiota has become a hot research topic in CVDs and metabolic diseases and is considered as the most promising interventive target of chronic diseases in the coming decades. Rapid developments and major breakthroughs in sequencing technologies have helped recognize the important role of the gut microbiota on body homeostasis, especially regarding obesity, insulin resistance, and CVDs. Animal and human experiments consistently showed that patients with hypertension had significantly lower gut flora richness compared with that of normotensive subjects (110). Two concurrent studies have reported the relationship between gut microbiome composition and hypertension (111, 112). A recent study by Mell et al. highlighted differences in the cecal microflora between salt-sensitive and -tolerant strains in Dahl rats (111). Moreover, changes in the variety and abundance of several short chain fatty acids in the plasma were observed after cecal transplantation. Thus, there is reason to believe microbial composition may affect the levels of short chain fatty acids in the plasma *via* roles in metabolism. Significant dysbiosis due to decreased microbial richness, diversity, homogeneity, and increased Firmicutes/Bacteroides ratios in hypertensive animals have been observed by comparing changes in the fecal microflora in spontaneously hypertensive and chronic Ang II-infused hypertensive rats (112). Another difference between the microbiota of normotensive and hypertensive patients is the production of LPS. *Veillonellae* are enriched in gut microbes in human hypertensive patients and produce LPS. Biofilms encapsulate bacterial communities in an extracellular matrix produced by bacteria, increasing their adhesion, and creating an environment that is more suitable for their grow. LPS-producing bacteria in biofilms were more inflammatory than Gram-negative bacteria were without biofilm protection (113, 114). This shows that the hypertensive microbiota are more inflammatory throughout the body and, because of the arrival of LPS in the brain, are more responsible for the possibility of ANS activation and hypertension.

## 5 Immunity in hypertension

**Figure 2:**
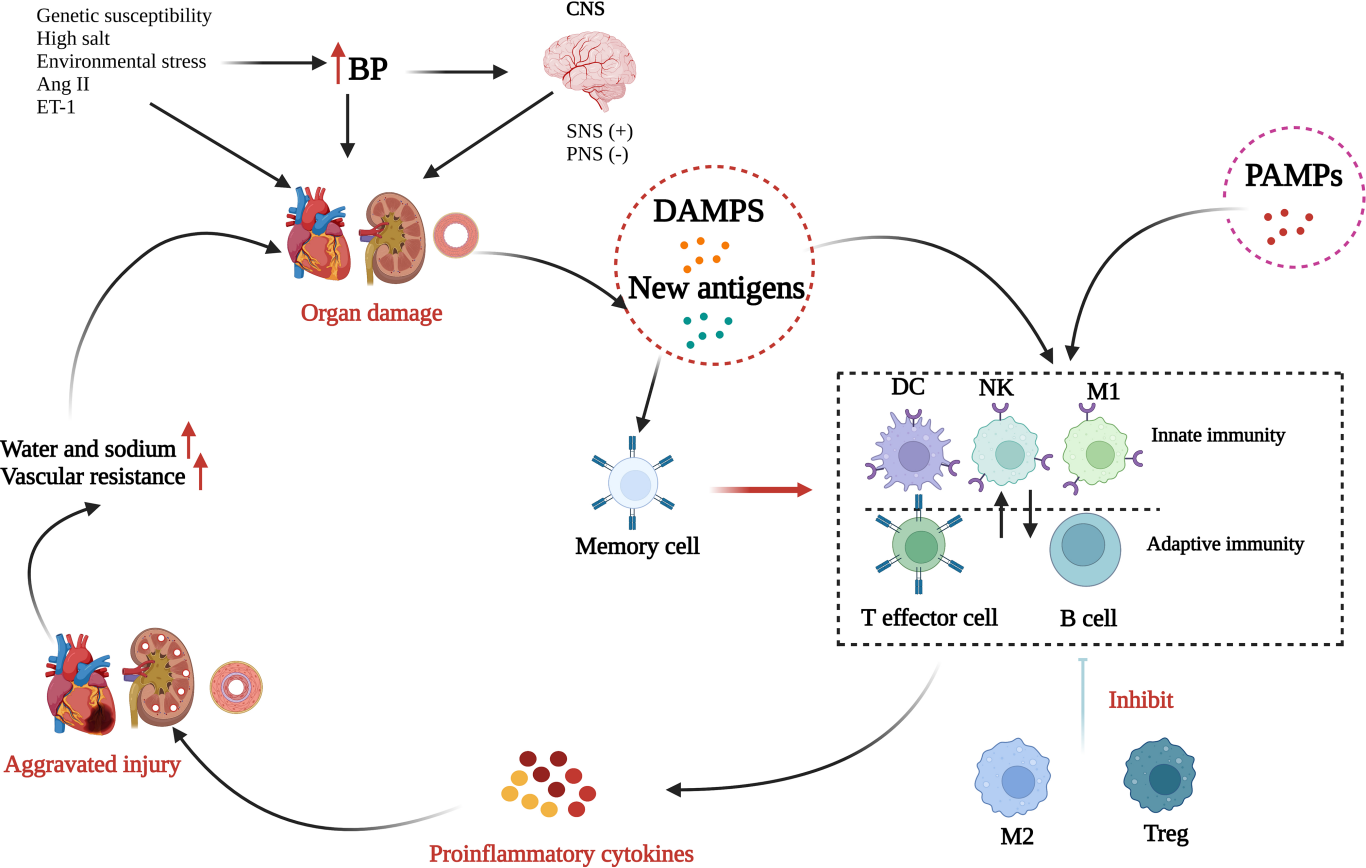
Role of inflammation and immunity in hypertension. Many risk factors, such as genetic susceptibility, high salt, and environmental stress, can elevate blood pressure (BP) and consequently activate the sympathetic nerve system (SNS) or inhibit the parasympathetic nerve system (PNS). Over time, elevated BP can induce organ injury promoting the formation of damage-associated molecular patterns (DAMPs) and new antigens. In addition, several infectious diseases can enhance the activation of the innate immune system through the binding of pathogen-associated molecular patterns (PAMPs) to toll-like receptors. DAMPs, new antigens, and PAMPs activate innate immunity which interacts with adaptive immunity. Next, numerous proinflammatory cytokines are released and result in further organ injury. Through alternatively activated macrophages (M2) and Tregs have roles in anti-inflammatory response, the effect is too weak to reduce the organ damage. Finally, peripheral vascular resistance and blood volume increases to result in hypertension (Created with BioRender.com).

The human immune system includes the IIS and the adaptive immune system (AIS), and the components of these function in an extremely complex and complementary manner to exterminate invaders and rescue injured tissue, thereby maintaining homeostasis. Most immune cells become involved in the initiation, progress, and maintenance of hypertension (**Figure 2**). Briefly, in the presence of genetic susceptibility, several environmental factors, especially salt and environmental stress, can cause a small rise in arterial blood pressure responsible for activating sympathetic nerves or inhibiting parasympathetic nerves. Next, elevated blood pressure causes or aggravates tissue damage and together with oxidative stress damage caused by Ang II and ET-1 is conducive to the formation of DAMPs and new antigens, and thereby initiates subsequent inflammatory activation. DAMPs trigger the activation of the IIS by recognizing and activating the Toll-like receptors (TLRs) on antigen-presenting cells while the new antigens enhance the immunogenicity of dendritic cells and facilitate their production and release of proinflammatory cytokines, which not only enable the proliferation and activation of T cells but also further produce more cytokines. DAMPS and new antigens can activate γδ T cells, which can activate T lymphocytes directly. Several infectious diseases can also enhance the activation of the IIS in a PAMP-dependent manner. Innate immune cells and γδ T cells release proinflammatory cytokines as well as autoantibodies, directly or through the activated AIS, producing vascular and renal damage, which is a feedforward process contributing to a progressive raise in blood pressure. In addition, additional effects, such high salt intake, may reactivate the T effector memory cells in the lymphoid organs, which will induce a more intense inflammatory response and cause more severe tissue damage. M2 macrophages and Treg cells have a role in anti-inflammatory response during this process, although their effect is very weak and not enough to reduce the organ damage.

### 5.1 Innate immune system

Innate immunity is the rapid, nonspecific defense response to a variety of exogenous stimuli that is often considered as the early stage of inflammation. The key effectors are mainly monocytes, M1 macrophages, natural killer cells, and neutrophils. Monocytes/macrophages have been revealed to be tightly associated with the microvascular remodeling. In a rodent model with a deficiency in macrophage colony stimulating factor, Ang II failed to induce endothelial injury and vascular oxidative stress or elevate blood pressure (115). Consistently, removing lysozyme M-positive myelomonocytic cells prevented mice from suffering severe hypertension (116). As mentioned above, macrophages are crucial to the progress of hypertension and, although the exact mechanisms remain elusive, increasing evidence indicates that a large amount of ROS derived from activated macrophages may cause irreversible vascular endothelial cell damage and microvascular remodeling, causing increased peripheral arteriolar resistance (116, 117). Experiments have shown that activated monocytes can produce endothelial dysfunction. Chemokines become involved in the pathological process of hypertension by recruiting immune cells to the site of endothelial injury, causing further impairment of endothelial function. An increase in the number of monocytes and neutrophils with the CXCL1 receptor CCR2 can be clearly detected in patients with hypertension, suggesting that blocking CCR2 may alleviate inflammation and control blood pressure. Neutrophils are an important component of the IIS and produce large quantities of cytokines and superoxide anion when stimulated (118). Ang-II could connect thromboinflammation with essential hypertension by inducing neutrophil to release extracellular traps. The use of plasma from untreated patients with hypertension to stimulate neutrophils in normal patients can facilitate the production of endothelial collagen, thereby causing vascular remodeling and injury (119). More recently, the gene *SH2B3 (LNK)* that encodes SH2B adaptor protein 3 have been confirmed that regulate the immune cell (notably T cells and macrophages) development, differentiation, and signaling in the hypertension according to the genome-wide association studies (120). Therefore, elucidating molecular mechanisms where *SH2B3* regulating immune cells in hypertension may found novel therapeutic targets for hypertension.

### 5.2 Adaptive immune system

Increasing evidence suggests that the activated AIS promotes the pathogenesis of hypertension and aggravates targeted organ damage. The AIS executes a highly specific response and is regulated by activated T and B lymphocytes. According to specific markers on the cells, T lymphocytes are divided into two subpopulations: CD4^+^ and CD8^+^ T lymphocytes. Although it has been confirmed that both types of T cells are involved in the initiation and progression of hypertension, results based on *in vivo* models showed that CD4^+^ T cells are likely to the key factor in promoting hypertension (121). However, Trott et al. reported that CD8^+^ T cells can be a crucial motivating force in experimental hypertension. Once the endogenous or exogenous antigens presented by antigen-presenting cells are recognized, lymphocytes are activated and differentiated into T effector cells or Treg cells (122). Under the action of inflammatory cytokines or chemokines, these activated T lymphocytes target the site of inflammation. The balance among T cell subsets can influence inflammatory responses. Kassan et al. (123) described the relationship between Tregs and vascular dysfunction in patients with hypertension and found that endothelial damage, plaque rupture, and arterial occlusion mainly resulted from the imbalance of Treg cells.

The hypothesis that T lymphocytes are the important participant in the pathogenesis of hypertension was proposed many years ago but failed to receive enough attention initially. It was not until the past 20 years that experimental evidence confirmed the exact role of T lymphocytes in elevating blood pressure and promoting vascular injury (76). Elevated blood pressure may further activate the immune response *via* modifying self-antigens or generating new antigens. Studies have shown that several T lymphocyte subsets may promote the occurrence of hypertension and vascular remodeling (124). T- and B-lymphocyte-deficient mice, produced by silencing recombination-activated gene 1 (RAG1^−/−^), exhibited a blunted hypertensive response compared with that in control mice in the Ang II-induced hypertension animal model. Adoptive transfer of effector T cells, but not B cells, to the RAG1^−/−^ mice restored the effects of Ang II (76). Moreover, in mice lacking T cells, there was insufficient infiltration of innate immune cells in vascular pathological sections, which was possibly related to the absence of cytokines derived from Th cells (76).

Studies have demonstrated that T lymphocyte-mediated immune responses can be induced by oxidative stress. The role of Th17, an important part of effector T cells, in hypertension has been identified by Madhur et al. (125). Th17 not only promotes but can also inhibit inflammation. Using the Ang II-induced hypertension animal model, improved vascular function, lowered oxidative stress levels, and decreased T lymphocyte infiltration have been observed in IL-17^−/−^ mice compared with these parameters in control mice (125). Madhur et al. revealed that the blood pressure level is positively correlated with the amount of circulating Th17 cells and that the inhibition of IL-17 contributes to the improvement of hypertension, which is consistent with previous research. In addition, enhanced acquired immunity due to genetic susceptibility, and vascular inflammation due to decreased Treg immunosuppressive function may contribute to hypertension (126). Viel et al. proposed a famous hypothesis that the genetic predisposition based on loci on chromosome 2 where many proinflammatory genes locate enhances adaptive immunity (127).

## 6 Oxidative stress in hypertension

Oxygen molecules can easily form free radicals because of their special electronic arrangement structure. These oxygen molecules are called oxygen free radicals (OFR) and include the superoxide anion, hydroxyl radical, and NO free radical. The hydroxyl radical is the most active OFR found so far. Furthermore, ROS, which includes H_2_O_2_ and O_3_ as well as OFR, is a critical signaling molecule that mediates the activation of transcription factors, induction of immune response genes, and the phosphorylation of kinases (128). Cumulative evidence from humans and animals suggests that ROS plays an important part in regulating endothelial cell function and vascular remodeling (129, 130). In the development of hypertension, the interaction of ROS and humoral factors such as ET-1 and Ang-II increases the production of ROS, especially the superoxide anion produced by the uncoupling of NOXs family and eNOS (130). Recently, studies have suggested that hypertension is associated with the decrease in NO and the increase in oxidative stress (131). ROS directly inhibits the activity of NO (132). In addition, ROS can stimulate the PI3K/Akt-MAPK pathway related to redox transcription factors causing overexpression of redox genes and thereby inhibiting the expression of eNOS mRNA and eNOS activity, which reduces the availability of NO. NADPH oxidase is regarded as the most important provider of ROS in vascular walls and endothelial cells and has an indispensable role in the pathogenesis of endothelial dysfunction and vascular remodeling. There is another crucial source of eNOS, xanthine oxidase and mitochondrial uncoupling, that helps to explain the increase in ROS production in different vascular diseases (133, 134). Previous evidence demonstrated that ROS regulates the arrangement of various proteins and the role of signal pathways in cells and that this redox biology is precisely and spatially regulated to influence the individual healthy conditions. Moreover, disordered physiological ROS production can cause a variety of diseases, including hypertension. During hypertension, neurohumoral factors can stimulate receptors located on the cell membrane to activate NADPH oxidase and mitochondria to produce ROS, such as highly active superoxide anions, which can then initiate cellular phosphorylation pathways (135). This subsequently initiates gene expression of factors such as p53, activating protein-1 (AP-1), nuclear E2-related factor 2 (Nrf2), and NFκB. Eventually, these changes result in endothelial dysfunction and hypertension (136, 137).

## 7 Novel potentially therapeutic strategies in hypertension

**Figure 3:**
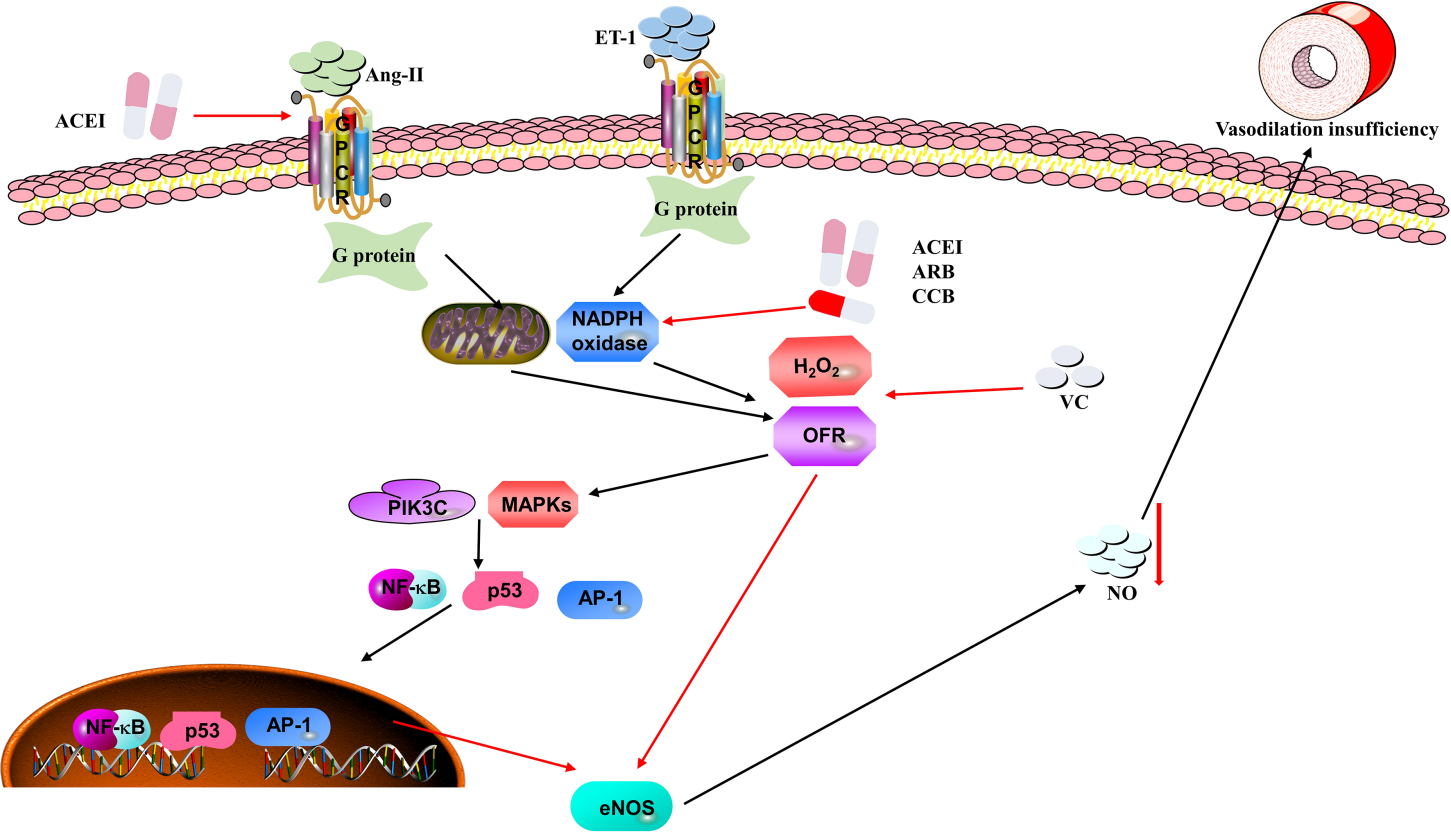
Mechanisms of oxidative stress causing hypertension in vascular endothelial cells. Ang II and ET-1 can stimulate NADPH oxidase and mitochondria to produce ROS (e.g., H2O2 and OFR), which are recognized by their receptors on the endothelial cells. ROS stimulate the PI3K/Akt-MAPK pathway to inhibit the expression of eNOS mRNA and eNOS activity, thus reducing the availability of NO, which results in vasodilation insufficiency and elevation of blood pressure. Moreover, angiotensin converting enzyme inhibitors (ACEIs), angiotensin receptor blockers (ARBs), calcium channel blockers (CCBs) and vitamin C (VC) may be potential therapeutic strategies in the process of oxidative stress.

Multiple animal experiments have proved that an insufficiency of vascular endothelial cells is closely related to the increase in arterial blood pressure. Considering the pathogenesis, prevalence, and severity of hypertension, it is critical to find novel and potential therapeutic strategies to lower blood pressure. In this section, we will summarize and explore the role of anti-inflammation and antioxidant therapies for hypertension (**Figure 3**).

### 7.1 Anti-inflammation therapeutic role in hypertension

As mentioned above, chronic inflammation has a close association with elevated blood pressure. Clinical studies indicate that elevated CRP levels at baseline are more likely due to an increased risk of hypertension (138). Although inflammatory activation is closely associated with elevated blood pressure, so far there is no obvious data supporting that routine use of anti-inflammatory drugs can treat hypertension. **Table 2** describes several drugs that may lower blood pressure *via* an anti-inflammatory effect. A preliminary analysis of one clinical study shows that minocycline, a broad-spectrum tetracycline antibiotic has a continuous antihypertensive effect in patients with hypertension (139). Mycophenolate mofetil is a well-known immunosuppressant, whose active metabolite inhibits the activation and synthesis of T and B lymphocytes by inhibiting dihydrolactate dehydrogenase and thereby decreases the synthesis of pyrimidine nucleotides, which can significantly lower blood pressure in salt-sensitive hypertension (140). In addition, a significant effect of mycophenolate mofetil on lowering blood pressure has been observed in patients suffering from psoriasis and rheumatoid arthritis (141). Another observational study showed that long-term use of immunosuppressive drugs can alleviate arterial stiffness and lower blood pressure in patients with chronic kidney disease (142). A monoclonal antibody against IL-17A could lower baseline blood pressure in patients with psoriasis (143). In addition, an IL-1 receptor antagonist (anakinra) could significantly reduce the blood pressure after a 14-day treatment *via* specifically inhibiting the actions of IL-1α and IL-1β (145, 146), but no significant antihypertensive effect was observed with canakinumab (an IL-1β antagonist) in large-scale randomized controlled trials in patients with previous myocardial infarction and elevated hsCRP (147). The study suggests that the mechanisms of reducing major adverse cardiovascular events are not associated with the change of blood pressure. The main reason that canakinumab does not lower the blood pressure may be related to the enrolled populations of whom 80% are patients with hypertension and were taking antihypertensive drugs, which may mask the real antihypertensive effect of canakinumab. Statins are widely used lipid-controlling drugs in clinics and can significantly reduce cholesterol levels, thereby reducing the risk of CVDs and improving prognosis. Statins also show multiple effects and have particular benefits on the cardiovascular system. Mounting evidence has demonstrated that statins have mild antihypertensive effects, especially in patients with intractable blood pressure and with hypertensive target organ damage (102, 144). The underlying mechanism whereby statins reduce blood pressure may be associated with the protective effect on the vascular endothelium by inhibiting the production of ROS, reducing the circulating level of proinflammatory cytokines, and inhibiting the expression of adhesion molecules on vascular endothelial cells and smooth muscle cells.

**Table 2:** Anti-inflammation therapeutic strategies in hypertension.

Agents	Intervention objects	Mechanisms	Biological effects	Reference
Minocycline	A patient with resistant hypertension	Has direct effects on gut microbiota	May cause a large reduction in Firmicutes and Bacteroidetes and a corresponding dramatic increase in Proteobacteria	(139)
Mycophenolate mofetil	Ang-II induced hypertensive rat models or patients with autoimmune disease	Prevents the development of salt-sensitive hypertension	Significantly reduces tubulointerstitial injury, superoxide-producing cells and T-cell infiltration and activation	(140–142)
Anti-IL-17A antibody	Hypertension rat models or patients with psoriatic arthritis	Inhibits IL-17 receptor unit A	Blocks the transport pathway including sodium hydrogen exchanger 3, Na-K-2Cl-cotransporter, Na-Cl cotransporter, epithelial sodium channel of renal tubules stimulated or upregulated by IL-17A	(72, 85–87, 143)
Statins	Patients with intractable hypertension	Anti-inflammatory and antioxidant effects that could reduce arterial stiffening and protect vascular endothelium	Inhibits the production of ROS, reducing the circulating level of pro-inflammatory cytokines and inhibits the expression of adhesion molecules on vascular endothelial cells and smooth muscle cells	(102, 144)

### 7.2 The value of antioxidant therapy in hypertension

Antioxidants are substances that effectively trap ROS, so they can reduce oxidative damage and may lower blood pressure (**Table 3**). The use of antioxidants, such as vitamin C and E, have been confirmed as effective therapeutic strategies in lowering the level of blood pressure. Vitamin C as an enzyme manager, increases eNOS activity and reduces ROS, and has been proved to improve vasodilation in hypertensive patients (158). Studies from humans and animals have shown that vitamin C can enhance endothelial function in a variety of situations (159). The effect of antioxidants on lowering blood pressure and enhancing vascular function has been confirmed in the hypertensive animals (160). One possible reason is that the free contractile response to norepinephrine is increased in patients with hypertension, which can be reduced by vitamin C (148). However, in several large-scale clinical trials, the effect of supplementation of vitamin C on blood pressure was unpredictable and more experiments are needed to confirm the exact effect (161–163), which may result from the reduced bioavailability of NO. B-cell lymphoma 6 (BCL6) is known as a key intervention target for autoimmune diseases by inhibiting production of ROS and apoptosis (149); however, it is unclear whether this can reduce hypertension. Chen et al. recently reported that BCL6 can lower blood pressure by suppressing vascular smooth muscle cell proliferation, attenuating oxidative stress injury, and microvascular remodeling in the Ang-II induced hypertensive rats (150).

**Table 3:** Antioxidant therapy in hypertension.

Agents	Intervention objects	Mechanisms	Biological effects	Reference
Vitamin C	Patients with hypertension	Have a beneficial effect on vasodilation	Antagonizing the free contraction induced by norepinephrine by antioxidation	(148)
BCL6	Ang-II induced hypertensive rats	Reduces producing of ROS and cell apoptosis	Inhibits vascular smooth muscle cells proliferation, attenuating oxidative stress injury and microvascular remodeling	(149, 150)
ACEIs/ARBs	Diabetic rats or patients with coronary artery disease	Suppress the RAAS producing ROS	Effectively inhibit the activity of NADPH oxidase, improve the superoxide dismutase activity, prevent eNOS from uncoupling and enhance of NO activity	(149, 151–153)
Novel beta-blockers	Hypertensive patients	Protect endothelial function	Reduces the ROS production from inflammatory cells and the oxidation of LDL, improve endothelium dependent vasodilation performance, inhibit the activity and expression of NADPH oxidase and prevent eNOS uncoupling and promoting eNOS activity	(154–156)
CCBs	Human aortic endothelial cells	Prevent oxidized low density lipoprotein	Lower the ROS production to alleviate oxidative damage	(157)

ACEIs, angiotensin converting enzyme inhibitors; ARBs, angiotensin receptor blockers; BCL6, B-cell lymphoma 6; CCBs, calcium channel blockers; eNOS, endothelial nitric oxide synthase; LDL, low density lipoprotein; NADPH, nicotinamide adenine dinucleotide phosphate; NO, nitric oxide; ROS, reactive oxygen species.

In addition, several antihypertensive drugs currently used in the clinic have a significant and specific effect on decreasing the incidence of hypertension independent of the expected mechanism. Next, we will mainly discuss the ACEI, ARB, beta-blockers, and CCBs. RAAS has a crucial part in the production of ROS during the process of hypertension. NOXs are recognized as one of most important sources of ROS in the endothelium and are mainly induced by Ang-II (149). Both ACEI and ARB can not only effectively inhibit the activity of NOXsbut improve the superoxide dismutase activity (151). In addition, they can prevent eNOS from uncoupling and enhance NO activity (152, 153). The novel beta-blockers, nebivolol and carvedilol, have been reported to have antioxidant properties. According to the results from clinical and experimental studies, these drugs have favorable protective effects on endothelial function and do not depend on the activity of beta-blockers. The antioxidant effect of carvedilol is mainly based on reducing the ROS production from inflammatory cells and the oxidation of low-density lipoprotein (LDL). Carvedilol and nebivolol could improve endothelium dependent vasodilation performance in patients with hypertension (154). Moreover, nebivolol exhibits a protective effect in the endothelial function and lowers blood pressure through suppressing the activity and expression of NADPH oxidase, thereby preventing eNOS uncoupling and promoting eNOS activity to produce more NO (155, 156). CCBs, especially dihydropyridines (like nifedipine) could directly reduce production of ROS to protect the endothelial function. CCBs have been demonstrated to prevent oxidized LDL, which can promote ROS production to trigger oxidative damage from causing endothelial dysfunction (157). Therefore, in addition to the direct effect of lowering blood pressure, dihydropyridine, CCBs play an additional antihypertensive effect by improving the function of vascular endothelial cells through antioxidation.

## 8 Conclusion

Accumulating evidence shows that hypertension is a chronic inflammatory condition that involves the migration, accumulation, and activation of immune cells and the production of ROS. Although many studies have been performed, the specific molecular mechanisms of inflammation and immunity that affect elevated blood pressure remain unclear. Inflammatory or oxidative stress can damage vascular endothelial cells and cause microcirculation remodeling; however, whether this can increase blood pressure through other mechanisms requires more research. Although there are many drugs that can treat hypertension, a deeper understanding of the mechanism of elevated blood pressure and the discovery of more action targets will be more conducive to earlier detection and intervention of hypertension and thereby significantly reduce the occurrence of cardiovascular adverse events. As emphasized in this review, the occurrence of hypertension is complex, so it is necessary to understand the pathological mechanism of the different stages. In addition, there is also an urgent requirement for the research and development of new antihypertensive drugs for different targets.

## Author contributions

(I) Conception and design: All authors. (II) Administrative support: None. (III) Provision of study materials or patients: None. (IV) Collection and assembly of data: None. (V) Data analysis and interpretation: None. (VI) All authors contributed to the article and approved the submitted version.

## Glossary

**Table:**

ACEIs	angiotensin converting enzyme inhibitors
Ang II	angiotensin II
AP-1	activating protein-1
ARBs	angiotensin receptor blockers
BCL6	B-cell lymphoma 6
CCBs	calcium channel blockers
CCL-2	C-C motif chemokine ligand 2
CVD	cardiovascular disease
DAMPs	damage-associated molecular patterns
EDGF	endothelium-dependent vasoconstrictor factor
eNOS	endothelial nitric oxide synthase
ET-1	endothelin-1
H_2_O_2_	hydrogen peroxide
IL	interleukin
IFN-γ	interferon gamma
LDL	low density lipoprotein
M1	classic activated macrophages
M2	alternatively activated macrophages
MMF	mycophenolate mofetil
NADPH	nicotinamide adenine dinucleotide phosphate
NADPH-Ox	nicotinamide adenine dinucleotide phosphate oxidase
NFκB	nuclear factor kappa-B
NO	nitric oxide
PAMPs	pathogen-associated molecular patterns
PPRs	pattern recognition receptors
RAAS	Activated renin-angiotensin-aldosterone system
RAG1	recombination-activated gene 1
ROS	reactive oxygen species
Nrf2	nuclear E2-related factor 2
Th	T helper
TLR	Toll-like receptors
TNF-α	tumor necrosis factor α
TNFR	TNF-α receptor
Treg	T regulatory cells
VCDM	vascular cell adhesion molecule
